# Domain Knowledge-Based Evolutionary Reinforcement Learning for Sensor Placement

**DOI:** 10.3390/s22103799

**Published:** 2022-05-17

**Authors:** Mingxuan Song, Chengyu Hu, Wenyin Gong, Xuesong Yan

**Affiliations:** School of Computer Science, China University of Geosciences, Wuhan 430074, China; chuqinghan@cug.edu.cn (M.S.); wygong@cug.edu.cn (W.G.); yanxs@cug.edu.cn (X.Y.)

**Keywords:** sensor placement, evolutionary reinforcement learning, domain knowledge, combinatorial optimization

## Abstract

Reducing pollutant detection time based on a reasonable sensor combination is desirable. Clean drinking water is essential to life. However, the water supply network (WSN) is a vulnerable target for accidental or intentional contamination due to its extensive geographic coverage, multiple points of access, backflow, infrastructure aging, and designed sabotage. Contaminants entering WSN are one of the most dangerous events that may cause sickness or even death among people. Using sensors to monitor the water quality in real time is one of the most effective ways to minimize negative consequences on public health. However, it is a challenge to deploy a limited number of sensors in a large-scale WSN. In this study, the sensor placement problem (SPP) is modeled as a sequential decision optimization problem, then an evolutionary reinforcement learning (ERL) algorithm based on domain knowledge is proposed to solve SPP. Extensive experiments have been conducted and the results show that our proposed algorithm outperforms meta-heuristic algorithms and deep reinforcement learning (DRL).

## 1. Introduction

The safety of drinking water is essential to life. If pollution events occur, they can cause significant losses. For instance, compared to other cities in Michigan, lead-related water contamination events in Flint reduced fertility by 12 percent [1]. Eight heavy metals have been detected in the Xiangjiang River, an important drinking water source, which has extremely high ecological risks and may cause serious drinking water pollution incidents at any time [2]. To detect pollution incidents earlier, reducing pollutant detection time remains an urgent and strategic task.

An important approach to improving the efficiency of pollution monitoring is to build an early warning system (EWS) for drinking water resources. The EWS detects the pollutants in the water body with water quality sensors. The water quality sensor can sample the water body, analyze the pollution, and report the pollution incident in real-time through the network. The widespread deployment of pollution EWSs can effectively reduce the risk of pollution incidents. A key link in the design of an EWS in the water supply network (WSN) is to arrange the sensors. The main components of WSNs are static sensors, which can be used to detect various pollutants. Compared with dynamic sensors, their price is mature, the cost is low, and they are easy to detect, maintain, and replace. Water quality sensors are expensive (for instance, Hach Chlorine Analyzers cost RMB 3000 to 5000) and there are also costs for sensor placement and maintenance. With a limited number of sensors, the ideal sensor placement solution minimizes the potential impact of contamination events on public health. Thus, it is of great practical significance to study the sensor placement problem (SPP).

Different environmental matrices have affects on the placement of the sensors. Sensors are placed in the water for a long term, and it is easy to cause a short circuit to the sensors due to moisture. Therefore, it is necessary to choose sensors with high air-tightness qualities. The sealing method of the sensor determines its air-tightness. Among the various sealing methods, welding sealing, thermal sleeve sealing, vacuum nitrogen filling, and glue sealing are widely used. From the perspective of sealing effects, welding sealing is the best, and glue sealing is the worst. For sensors that work in a clean and dry indoor environment, it is suitable to use glue-sealed sensors. For most SPP, the working environment of the sensor is relatively humid, and you should choose diaphragm heat sealing or diaphragm welding sealing, for vacuum-charging nitrogen sensors. In addition, sensors in highly corrosive environments may have short circuits or damage to their elastomer due to humidity and acidity. The outer surface should be over-sprayed or a stainless steel cover, for corrosion resistance and air-tightness.

**Figure 1:**
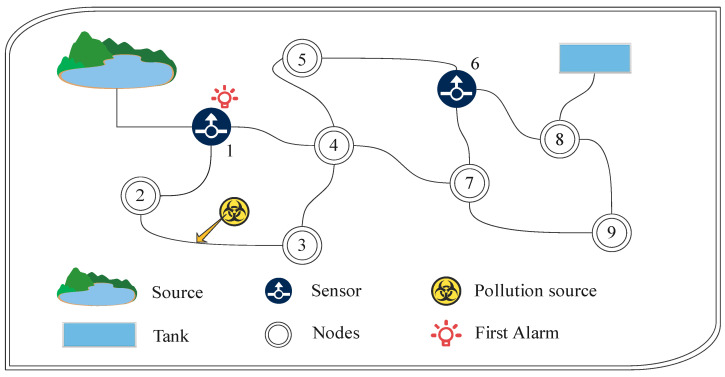
Layout of the water quality sensors in a small WSN (9 nodes, 2 sources, 1 tank, 13 pipes).

As shown in Figure 1, a typical SPP is given in a small WSN. There are nine nodes in the WSN, and the sensors are deployed at nodes 1 and 6. Specifically, if a pollution event occurs at the location in the figure, the sensor at node 1 can raise the alarm as soon as possible. Due to the large scale of WSNs, where to deploy the sensors is a great challenge. In essence, SPP is a kind of combination optimization problem, which have been proved to be NP-Complete [3]. Various sensor placement algorithms are proposed [4,5,6,7,8,9]. SPP can be formulated as a constrained optimization problem based on some model that captures the effect of different sensor combinations to minimize detection time. For instance, paper [4] solved SPP with a branch-and-bound algorithm and papers [5,6] use integer programming. However, when the scale of the WSN becomes larger, the solution time of the deterministic algorithm increases significantly or even cannot solve the problem. SPP can also be solved by the sorting algorithm. The authorities only need to provide accurate WSN information and the professional opinion of the SPP from relevant experts. Although the computational overhead of this method is small, it can also lead to inaccuracy due to human subjectivity [7,8,9].

Recently, deep reinforcement learning (DRL) has shown great ability in solving combinatorial optimization problems with an uncertain and dynamic environment. Combinatorial optimization, which is the optimal selection of decision variables in a discrete decision space, has naturally similar characteristics to reinforcement learning’s (RL’s) “action selection”. Furthermore, owing to the strong generalization ability of neural networks, it is possible for DRL to solve large-scale combinatorial optimization problems [10,11,12]. Thus, using DRL to solve combinatorial optimization problems is a good choice. In view of this, a series of new approaches to solve combinatorial optimization problems using DRL methods have emerged in recent years. Combinatorial optimization problems such as capacitated vehicle routing problems (CVRP) and graph coloring problems (GCP) have achieved good results. Lu H. [13] use transformer attention combined with the RL method to improve solution speed for CVRP. Emre Yolcu [14] used graph neural network (GNN) combined with RL method in GCP to get optimal solutions with fewer search steps. Compared with the traditional combinatorial optimization algorithm, the DRL-based combinatorial optimization algorithm has a series of advantages such as fast solution speed and strong generalization ability [15].

However, DRL algorithms typically suffer from three core difficulties: temporal credit assignment with sparse rewards, lack of effective exploration, and brittle convergence properties that are extremely sensitive to hyperparameters [16]. In order to further enhance the performance of DRL, evolutionary computation (EC) has been integrated into the framework of DRL, called evolutionary reinforcement learning (ERL) [17]. As a combination of EC and DRL, ERL inherits the advantage of EC and DRL, and it has several many attractive characteristics. First, it does not care about the distribution of rewards (sparse or dense). Second, gradient information will accelerate ERL, and population mutation and crossover operation can strengthen search ability. Section 5.2 presents our experiments on the performance of different algorithms in SPP. By comparison, ERL outperforms DRL.

In this paper, we take SPP in WSN as a sequential decision optimization problem and use ERL to solve it. The paper [18], uses the RL paradigm to solve SPP in distributed parameter systems, exploiting the time-space separation property to minimize the modeling error in the entire temporal and spatial domains. Differently from above, we focus on the pollution detection time in WSN and introduce a deep neural network based on the RL paradigm, combining EC and domain knowledge to solve SPP. If the traditional optimization algorithm is used to solve SPP, experiments on Section 5.2 showed that the effect was not good. By modeling SSP as a sequential decision problem, like greedy search instead of optimization, the effect is improved. For the ERL algorithm to solve SPP, the algorithm initializes a population where each individual contains a neural network. SPP is mathematically formulated as a Markov decision process (MDP) with specified elements. By splitting sensor placement actions, previously placed sensors are considered when selecting each sensor location. Then, through the individual’s own exploration and information sharing among individuals, the algorithm outputs the sensor combination with excellent detection effects. In contrast, the DRL algorithm lacks information sharing among individuals, and the individual exploration ability of the EC algorithm does not originate from the neural network. In order to further enhance the performance of ERL, domain knowledge is also integrated. The measurement of the average detection time on each sensor node for a set of pollution events consumes a small amount of computation but is closely related to performance. Therefore, we tested the detection time of each sensor individually for the pollution event set and injected the average detection time of each sensor location into the model as prior knowledge. Section 5.3.1 also has our performance experiments on ERL with domain knowledge. By comparison, ERL with domain knowledge outperforms DRL and meta-heuristic algorithms.

As mentioned above, the main contributions of this paper are as follows:

- SPP is modeled as a sequential decision optimization problem, a framework of ERL has been proposed to solve it. As far as we know, this is the first work to apply ERL to the SPP in WSN.
- To further enhance the performance of ERL, domain knowledge is employed to improve the search ability. Extensive experiments have been conducted and the results show that the proposed algorithm outperforms traditional heuristic algorithms and DRL.

The rest of this paper is structured as follows. Section 2 introduces the related work. Section 3 formulates the SPP and gives an overview of the algorithm. Section 4 introduces the framework of ERL algorithm based on domain knowledge and genetic algorithm. Section 5 discusses the experiment results. Finally, Section 6 shows the results and discussion of the paper.

## 2. Related Work

### 2.1. Sensor Placement Problem

SPP is currently one of the research hotspots for academia and industry. Due to the complex geographical distribution and numerous entry nodes, the WSN is vulnerable to accidental or man-made pollution injection attacks [19]. Thus, it is especially significant to place drinking water quality sensors in WSNs, as they can monitor and report contaminants in real time. Under ideal conditions, we can deploy sensors at each node of the WSN. Actually, the pipe network has complex geometries and topologies due to the connectivity of the network, the location of each single element (e.g., pipes, pumps, valves), and the complex network geometry produced by traditional design criteria [20]. Additionally, water quality sensors are expensive and there are also costs for sensor placement and maintenance. Considering the limits of geographic location and economic conditions mentioned above, only a limited number of sensors can be deployed in a WSN. Thus, reasonable layout of sensors can not only effectively reduce the cost, but also improve the efficiency of monitoring.

In general, academics divide SPP into two categories: the maximum coverage problem and the set coverage problem. The maximum coverage problem of SPP mainly refers to maximizing monitoring efficiency by a fixed number of sensors, and the set coverage problem of SPP is studied to achieve full coverage of the monitoring area through the minimum number of sensors. Because the size of WSN is greater than the number of sensors, the maximum coverage problem is studied in our paper. In addition to the classification of the problem, the size of the WSN will also have a great impact on the performance of different algorithms. When the size of the WSN is small or medium, deterministic algorithms, such as integer programming [21], branch and bound [22], and heuristic algorithms (e.g., genetic algorithm), are often used to solve the SPP. For instance, Jonathan Berry [5] presented a mixed-integer programming (MIP) formulation for sensor placement optimization in municipal water distribution systems. Zhao Y. [4] proposed a branch and bound sensor placement algorithm based on greedy heuristics and convex relaxation. However, as the size of WSN increases, the computational complexity increases exponentially, and neither deterministic algorithms nor heuristic algorithms can solve the SPP quickly and accurately.

In order to deal with large-scale SPPs, there are two methods. The first one is the expert opinion method, which allows experts in related fields to select the location of the sensor based on their experience. In the early days, this method was mainly adopted due to technical limitations. The drawback of the expert opinion method is that it relies too much on expert subjectivity, making the results inaccurate. The second one is the sorting method. The sorting method is a rule-based method, and the algorithm sorts the sensor placement positions by defining some rules. Thus, most of the sorting methods do not need to obtain models for hydraulic simulation and water quality simulation in the water supply network, and only need to calculate the priority of each node according to the rules. Thus, the sorting method can be used in the sensor arrangement of large-scale networks without a hydraulic model. Morais [7] developed a multi-criteria model for group decision-making to optimize WSN. Although this method has small computation overhead, it will also be inaccurate because of human subjectivity, and one rule does not apply to different WSN models (rings, tree structures).

Additionally, parallel computing is also effective for solving large-scale SPP problems. Parallel computing accelerates computing by using large-scale hardware resources, which can solve large-scale SPPs to some extent. For instance, Hu C. [23] used Map-Reduce to solve contaminant source identification problems in WSN. The simulation time can be greatly shortened through parallel computing. However, the SPP is essentially a combination optimization problem in a high-dimensional space. As the size of WSN increases, the decision space increases exponentially, and parallel computing will fail to solve SPP.

As the uncertainties are also key factors that have an impact on the monitoring effectiveness of sensors, the above methods do not consider the uncertainty of water demand and the randomness of pollution events [24]. The basic process of reinforcement learning is a Markov decision process, through the strategy of reinforcement learning, switching between different sensor placement states to tackle the SPP in a dynamic uncertain environment. Thus, we model SPP as a sequence decision optimization problem, and apply ERL to solve it.

### 2.2. Evolutionary Reinforcement Learning

With the proposal of AlphaGo [25], artificial intelligence-related algorithms have attracted widespread attention from the academic and industrial communities. The core of AlphaGo is DRL, which has been widely applied in the field of intelligent traffic control, computer games, robot control, and natural language processing. Furthermore, with the advent of pointer network architecture [26], DRL can deal with large-scale combinatorial optimization problems. For instance, the pointer network is applied to solve TSP and vehicle problems [27,28]. Chen X. [29] achieved better results on expression simplification, online job scheduling, and vehicle routing problems using a method named NeuRewriter with a pointer network.

There are also cases of DRL applications in WSN-related problems. For instance, Gergely Hajgató [30] used DRL to solve real-time control of pumps in WSN. DRL can also be used to solve the SPP. DRL has the ability in solving sequential decision optimization problems by using an agent interacting with the environment, and SPP can also be viewed as a sequential decision optimization problem. The layout of sensors is a series of actions to deploy the sensor one-by-one in a WSN. Before deploying each sensor, it is necessary to observe the environment state and decide to deploy the next sensor in a WSN. As the decision space of SPP is relatively complicated, it is appropriate to use a deep neural network for the output strategy, to select the optimal node to deploy the water quality sensor.

From the above analysis, it can be seen that DRL has achieved great success in a series of challenging combinatorial optimization tasks. However, DRL also has some drawbacks: temporal credit assignment with sparse rewards, lack of effective exploration, and brittle convergence properties that are extremely sensitive to hyperparameters [16]. Due to these three shortcomings, DRL cannot be widely applied to more practical problems. As shown in Section 5.2, it can be seen that there is still a certain gap between the effect of DRL and the meta-heuristic algorithm on SPP.

As a competitive optimization algorithm, EC can deal with many complex optimization problems without gradient information. EC consists of three main operators: new solution generation, solution change, and selection. These operations are applied to a population of candidate solutions to continuously produce new solutions while preserving promising probabilities. The selection operation is usually probabilistic, in which a solution with a higher fitness value has a higher probability of being selected. If a higher fitness value represents a good solution quality, the overall quality of the solution will improve with each generation. Mambretti [31] uses a method based on Genetic Algorithms to optimize the pumps functioning in water distribution networks.

By combining EC with RL, ERL inherits the advantage of EC and RL, and has the ability to deal with complex practical problems. In paper [32], Khadka used the population of the EC to provide diverse data to train the agent, and periodically reinsert the agent into the population. By sharing the gradient and hereditary information, ERL can allocate time credits through fitness measures, and effectively explore a set of different strategies. In our work, deep neural networks are used to replace individuals. By directly encoding the neural network layers as the chromosome, we evolve multiple chromosomes by crossover, mutation operations. At the same time, gradient information is also used to update the parameters of neural networks, acting as the selection operator.

Additionally, in many domains, the application of domain knowledge also provides better potential for model algorithms. Zhang K. [33] used domain knowledge to revise the results from the RL model so that their model achieved state-of-the-art results on most specific sentiment categories. Zheng Y. [34] used domain knowledge to improve the robustness of deep neural network models, reducing the model’s standard deviation by 37%. Many DRL algorithms contain inductive bias, which includes domain knowledge [34]. In general, there is a trade-off between generality and performance when algorithms use this bias. Stronger biases can lead to faster learning, but weaker biases can lead to more general algorithms. This may require a lot of effort to gain relevant domain knowledge or tune hyperparameters efficiently. In this paper, we carefully select the appropriate domain knowledge to incorporate it into the algorithm with almost no additional workload, achieving remarkable results on the SPP.

## 3. Problem Modeling and Approach Overview

This paper aims at developing an ERL-based node selection mechanism combined with domain knowledge to find optimal sensor combinations in WSN. This section states the problem and presents the overview of the proposed approach.

### 3.1. Problem Modeling

The urban WSN is modeled as a directed graph G=(V,E), which consists of nodes and edges, *V* means the set of nodes, and the sensors can be deployed at any node. *E* means the pipes in WSN. When a pollution event occurs, the pollutants will flow with the drinking water along the pipeline in the WSN. When the concentration of contaminant exceeds the detection threshold of the sensor, the sensor will raise an alarm in time.

There is a possibility that the sensors cannot detect pollution events. In this circumstance, the average minimum detection time is equal to the total simulation period *T*. It is assumed that the pollutants are conservative and do not react with water. Each pollution event involves a single injection location, that is, each pollution event occurs only at one node at any time with equal probability. In addition, we assume that the sensor can immediately detect any non-zero concentration of pollutants, and immediately raise an alarm.

We give definitions of states and actions to explain how to obtain a set of sensor nodes. First, the SPP’s system state *x* is as follows:

(1) $$x=\left[x_{1},x_{2},\ldots ,x_{i},\ldots ,x_{N}\right]$$

where *i* is an integer between 1 and *N*, and $x_{i}$ is equal to 0 or 1, representing whether the *i*-th node has placed a sensor. *N* is the number of positions where the sensor can be placed. The system state *x* is an N-dimension zero vector at the beginning, and continues to change from 0 to 1 with each node choose action, until the number of 1 reaches the number of sensor placements. In the final state, the dimension with a value of 1 is the node where the sensor needs to be placed, and the dimension with a value of 0 does not need to be placed.

Second, the node choose action is as follows:

(2) $$a=\left[a_{1},a_{2},\ldots ,a_{i},\ldots ,a_{N}\right]$$

where $a_{i}$ is equal to 0 or 1, and there is only one value equal to 1, representing the node position selected this time.

In this paper, we abstract each pollution event *W* as a set of *N* times, denoted as $W=\{t_{1},t_{2},\ldots t_{i},\ldots ,t_{N}\}$. Here, $t_{i}$ is the detection time of the sensor on the *j*-th node. For each pollution event *W* in the pollution event set, the detection time of the sensor combination is the time elapsed from the occurrence of the pollution event to the first determination of a non-zero pollution concentration by the sensor. For sensor node combinations, the minimum detection time is equal to the time when a pollution event is first detected across the chosen sensors. The minimum detection time for a specific pollution event is defined as follows:

(3) $$t_{n}=min\;\left\{t_{i}\right\}$$

where $t_{n}$ represents the minimum detection time among all *n* sensors placed in WSN, $t_{i}$ is the detection time of the sensor on the *i*-th node, and $\left\{t_{i}\right\}$ represents the set of times from the nodes placed in WSN. The objective function *J* in Equation (5) to be minimized is the expected minimum detection for multiple pollution events.

Thus, given a set of nodes, expressed as $S={\left\{s_{i}\right\}}_{i=1}^{n}$, where *n* is the number of sensors placed in WSN, the average minimum detection time for *m* pollution events in the pollution event set is defined as follows:

(4) $$t=\frac{1}{m}\;\overset{m}{\sum_{i=1}}\;min\;\left\{t_{ij},\;j=1\;to\;n\right\}$$

where $t_{ij}$ represents the minimum detection time of the sensor on the *j*-th node for the *i*-th pollution event.

By defining states and actions, following the standard formulation of RL, the allocation action *a* is chosen to achieve the function *J* defined in the Equation (5). This process is repeated until the target number of sensors is reached, and is described in more detail in Section 4. With the support of domain knowledge and evolutionary algorithms, this process can be better assisted. Finally, the selected sensor nodes are more accurate, and a group of sensor nodes with better results is finally obtained.

### 3.2. Approach Overview

First, we take domain knowledge as the input to the neural network. Then, the neural network of the model aims to learn the parameters of a stochastic policy to assign high probabilities to short detection times and low probabilities to long detection times. Then, our final objective *J* is defined as:

(5) $$J\left(\theta \;|\;V\right)=min\;E_{p(S|V)}\;t_{j}$$

where E denotes the mathematical expectation, and θ presents the parameters of the neural network in the model. In the Equation (5), p(S|V) is the chain rule to factorize the probability of a node combination, and more details are described in Equation (5) in Section 3.2.

In the current state *x*, the model selects the corresponding action, so that the system enters another state. The number of sensors selected in the current state is one more than the previous state. The size of the state-action space is $2^{n}\times N$, where *n* is the number of sensors placed in WSN, and *N* is the total number of nodes that can be placed. As *N* and *n* are often large in WSN, it is infeasible to precompute the action for every possible state to minimize the function *J* mentioned in Equation (5). However, SPPs have relatively simple reward mechanisms that could even be used at test time. Thus, using a model-free policy-based RL to optimize the parameters of a pointer network, and the specific update process are described in Section 4.

Finally, through the above process, the network can be updated after selecting a set of sensor combinations. The above only discusses the case of a single agent individual. When multiple agents form a population, the situation is more complicated. In the multi-agent case, we use DRL combined with EC to train the population. Each individual updates the individual through the above algorithm of implicit parallelism. Then, the population will evolve through crossover and mutation operators. The crossover operator exchanges the network layer parameters of some individuals with a certain probability, and the mutation operator resets the network layer parameters of some layers with a certain probability. After the above process, the individual has completed a round of evolution. The above process is iterated until the specified generation is reached. Section 4 shows some details of evolutionary algorithms, and our experimental results are shown in Section 5.

## 4. Domain Knowledge-Based Evolutionary Reinforcement Learning

**Figure 2:**
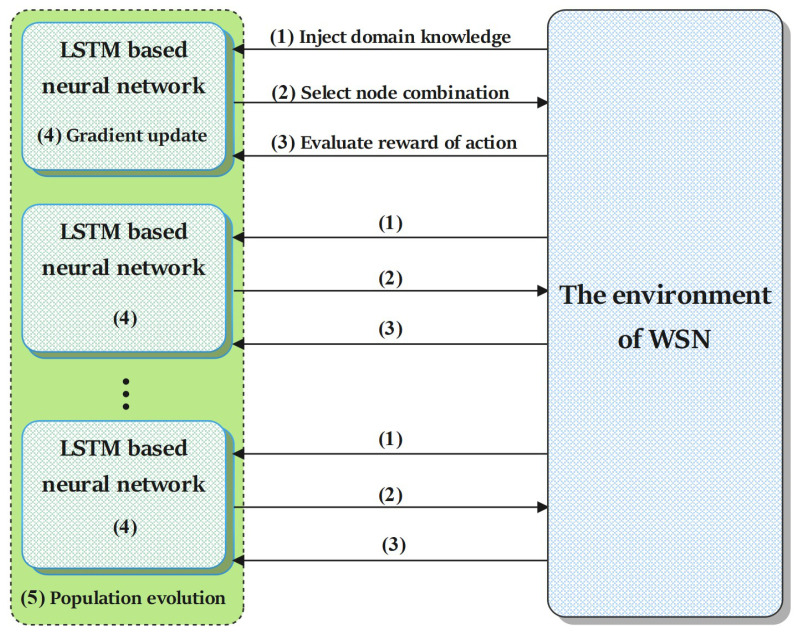
The framework of the domain knowledge-based ERL algorithm. Please note that the same serial number represents the same operation.

The overall framework of the algorithm is shown in Figure 2 and is described in detail in Algorithm 1. The details of each part of the algorithm are shown in the rest of this chapter.

### 4.1. Model Input with Domain Knowledge

It is worth noting that we consider using the Monte Carlo method to search extensively when generating the initial sequence, so that the model can learn from a better initial solution. However, experiments in Section 5.3.2 have shown that this approach is also not very effective. We try to use domain knowledge to overcome the problem. Because the detection time of a single sensor for all pollution events indicates, to a certain extent, the sensor’s monitoring ability, it will provide a certain contribution to the detection ability of the sensor combination. Thus, the input vector *I* to our neural network is defined as follows:

(6) $$M_{j}=min\left\{t_{ij},\;i\in 1\;to\;m\right\}$$

(7) $$I_{j}=1-\frac{M_{j}-M_{min}}{M_{max}-M_{min}}$$

where $I_{j}$ represents the *j*-dimension of the input vector *I*. Experiments in Section 5.3.1 show that this method can significantly improve search performance.
**Algorithm 1** Domain knowledge-based ERL**Input:** input nodes set *V*, initial parameters θ, the number of pollution incidents *m*, choose nodes number *n*, baseline shift coefficient α, population size *k*, iteration *l*, iteration *l*, crossover probability $p_{c}$, mutation probability $p_{m}$InitialDomainKnowledgeS←NodeChooseAction()$t_{S}\;\leftarrow \;t\left(S\;|\;V\right)$$b\;\leftarrow \;t_{S}$**for** it=episode∈[1,l]** do**   **for** j=episode∈[1,k] **do**     $S_{j}\leftarrow NodeChooseAction\left(\right)$ for i∈{1,…,n};     $j\leftarrow Argmin(p\left(x_{1}|V\right)\ldots p\left(x_{n}|V\right))$;     $t_{j}\leftarrow t\left(S_{j}|V\right)$     **if** $t_{j}$ < $t_{S}$ **then**        $S\leftarrow S_{j}$        $t_{S}\leftarrow t_{j}$     **end if**     $g_{\theta }\leftarrow \frac{1}{m}\sum_{i=1}^{m}\left(t_{j}-b\right)\nabla_{\theta }log\;p_{\theta }\left(S\;|\;V\right)$     $\theta \leftarrow SGD(\theta ,g_{\theta })$     $b\leftarrow \alpha \times b+\left(1-\alpha \right)\times \left(\frac{1}{n}\sum_{i=1}^{n}b_{i}\right)$   **end for**   **for** $k\;=\;episode\in [1,\left\lfloor \frac{k}{2}\right\rfloor ]$ **do**     $\gamma_{1}=random\left[0,1\right]$     **if** $\gamma_{1}<p_{c}$ **then**        Crossover     **end if**   **end for**   **for** j=episode∈[1,k] **do**     $\gamma_{2}=random\left[0,1\right]$     **if** $\gamma_{2}<p_{m}$ **then**        Mutation     **end if**   **end for****end for****Return** *S*

### 4.2. Node Choose Action for Reinforcement Learning

Our pointer network consists of one layer of 128-unit fully connected linear network modules and two recurrent neural network (RNN) modules, which called the encoder and the decoder. Both RNN modules composed of 128 long short-term memory (LSTM) units. The input vector *I* in Equation (7) is linearly transformed by the feedforward network layer, and then transformed into a potential memory state sequence $\left\{enc_{i}\right\}$ by the encoder. At time step *i*, the decoder network maintains its latent memory state $\left\{dec_{i}\right\}$, and uses a pointing mechanism to generate the next sensor node to be selected in the combination. The computations of the pointing mechanism are parameterized by two attention matrices $W_{ref},W_{q}\in R^{d\times d}$ and an attention vector $v\in R^{d}$ as follows:

(8) $$u_{i}=\left\{\begin{array}{c} v^{T}\cdot tanh\left(W_{ref}\cdot r_{i}+W_{q}\cdot q\right),\;if\;i\neq \pi \;\left(j\right)\;for\;all\;j<i\\ -\infty ,\;\;\;\;\;\;\;\;\;otherwise \end{array}\right.$$

(9) $$A\left(ref,q;W_{ref},W_{q},v\right)\overset{def}{=}softmax\left(u\right)$$

Our pointer network, at decoder step *j*, then assigns the probability of visiting the next point π(j) of the combination as follows:

(10) $$p\left(\pi \left(j\right)|\pi \left(<j\right),s\right)\overset{def}{=}A\left(enc_{1:n},dec_{j}\right)$$

When generating sensor nodes, the pointer maintains a mask array to ensure that previously output nodes are not output. The output of the pointing mechanism is the probability of different nodes being selected in the current state, so there are multiple node choose strategies.

Aiming to learn the parameters of a stochastic policy p(x|V). With the learned policy, given an input set of nodes V, the model assigns high probabilities to short detection time and low probabilities to long detection time. Our neural network architecture uses the chain rule to factorize the probability of a node combination as:

(11) $$p\left(x\;|\;V\right)=\prod_{i=1}^{n}p\left(x\left(i\right)\;|\;x\left(<i\right),\;V\right)$$

where x(<i) presents the previous state of x(i) and *n* is the number of sensors placed in WSN, then uses individual softmax modules to represent each term on the source value on the right side of Equation (11).

However, due to the computational cost of computing with the reward being small, we improved the node selection strategy and used probabilistic selection, so that the results of the model are not unique for the same network, and the model can be used in reasoning to simulate the search process by considering multiple candidate solutions and selecting the best solution. The solver searches for a large set of feasible solutions each time to implement the inference process. Experimental results show that this approach significantly improves search results. Once the next sensor node is selected, it is passed as input to the next decoder step. The input to the first decoder step is a d-dimensional vector, which is treated as a trainable parameter for our neural network. Finally, the model outputs a set of nodes, expressed as $S={\left\{s_{i}\right\}}_{i=1}^{n}$, where *n* is the number of sensors placed in WSN.

### 4.3. Reward for Model Update for Reinforcement Learning

Vinyals [26] uses a supervised loss function containing conditional log-likelihood to train the pointer network. This function takes the cross-entropy target between the output probability of the network and the target provided by the solver into consideration. However, learning from examples in this way is not advisable for all NP-hard problems, especially SPP. SPP is untagged, and people are more concerned about finding a competitive solution without tags. Thus, when training the pointer network, instead of using the supervised loss function, we still considered the conditional log likelihood as an influencing factor of the gradient update.

To achieve our final objective described in Equation (5), the algorithm resorts to policy gradient methods and stochastic gradient descent to optimize the parameters. For the purpose of self-attention, we choose the exponential moving average as the gradient. The gradient of J(θ|V) is formulated as follows:

(12) $$\nabla_{\theta }J\left(\theta \;|\;V\right)=E_{p(S|V)}\left[\left(t_{j}-b\right)\nabla_{\theta }log\;p_{\theta }\left(S\;|\;V\right)\right]$$

where $t_{j}$ is shown in Equation (4). The *b* is an exponential moving average value of the rewards obtained by the network over time, accounting for the fact that the policy improves with training, and updates as follows:

(13) $$b_{j}^{\prime }=b_{j}*\alpha +t_{j}*\left(1-\alpha \right)$$

The model needs to minimize the detection time, so we take the difference between the detection time and *b* as a part of the reward. When the time of this iteration is small, the $\left(t_{j}-b\right)$ term is negative to achieve a local minimum search. The model loss value is the logarithmic result of multiplying the above expression by the gradient. Using a gradient descent method with the loss value, the model actively updates its parameters while searching for candidate solutions on a single test instance.

### 4.4. Evolutionary Strategy

The evolutionary strategy is a heuristic search process inspired by natural evolution: populations are perturbed at each generation, and their fitness value is evaluated accordingly. The parameter vectors with the highest scores are then recombined to form the next generation population. This process iterates until the goal is fully optimized. The difference between types of evolutionary algorithms is how they represent populations and how they perform mutation and recombination.

We first combine evolutionary algorithms and DRL individuals, set the population number as *n*, and realize it by searching multiple individuals in parallel. There is no communication between individuals in the population. However, the experiments in Section 5.3.3 show that this only slightly improves the detection time of the combination of nodes. Therefore, we adopt measures similar to genetic algorithms to update and reproduce the population to obtain the next generation of individuals.

The evolution operator in the model includes two parts: crossover operator and mutation operator. The crossover operator of the model directly changes the hidden layers of neural network, and due to the close correlation between the different network layers of the neural network, we randomly exchange all the parameters held by the first *n* layers of the network layer, so that both the performance of the deep neural network, and the evolutionary performance of the evolutionary algorithm. The mutation operator of the model randomly resets all the parameters of a layer of the neural network. After performing population evolution, the parameters of the neural network are changed. The model re-outputs sensor node combinations based on the set of pollution events and evaluates the effect of the new solution quality.

It is worth noting that there are not operations for the selection operator because when the agent of the deep neural network outputs actions, the selectivity of the model is improved by considering multiple candidate solutions based on the probability selection and batch in parallel. Therefore, the model already includes the effect of the selection operator. Experiments in Section 5.3.3 show that after adding the EC part, the experimental results outperform traditional meta-heuristic algorithms.

## 5. Experiments

### 5.1. Experimental Setup

**Figure 3:**
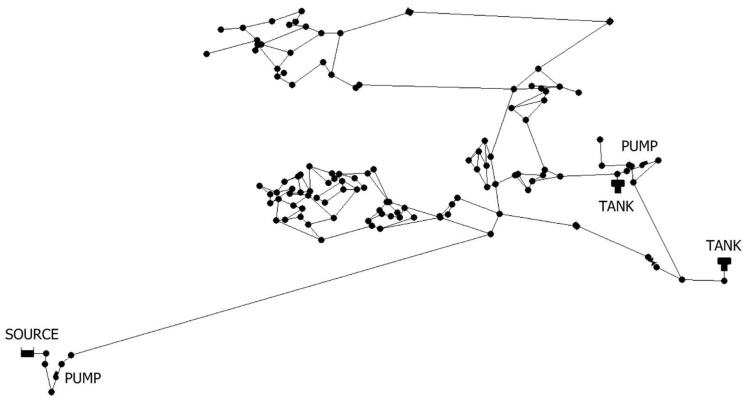
The network researched in the experiment.

A typical WSN shown in Figure 3 is employed in our experiments. The WSN consists of 126 nodes, 1 source, 2 tanks, 168 pipes, 2 pumps, and 8 valves. The time step in the simulation is 5 min and the whole simulation period is 96 h.

In order to evaluate the performance of our proposed algorithm, we compared the modified ERL to the DRL and genetic algorithm (GA). The hyperparameters of all algorithms are set as follows:

In GA, the length of the chromosome gene is the number of sensors, and the value of each gene is the number of the node in which the sensor is deployed. In the reproducing operation, the first half of the parents with the higher fitness values are selected as the next generation, and the multiple point crossover operator and single point mutation operator are used to generate the offspring. The probability of crossover is set as 0.6, and the probability of mutation is set as 0.1. When the population evolves 500 generations, the algorithm stops.

In DRL, a mini-batch size is defined of 512, and the search step length is set to 500 steps, which is consistent with the GA. A larger learning rate of 1 × 10${}^{-2}$ is set to ensure model convergence. Stochastic gradient descent (SGD) optimizer is used to update the parameters of network. Parameters are initialized uniformly and randomly in the range of (−0.8,0.8), and the L2 norm of the gradient is clipped to 1.0. The baseline attenuation is set to α = 0.99.

In ERL, each of our individuals is an agent, which is based on the network described in the DRL. The details of each individual are exactly the same as in DRL, and the crossover and mutation probabilities are also set as 0.6 and 0.1, respectively. For the ERL algorithm, we performed a sensitivity analysis to explore the effect of each part of the algorithm. It is worth noting that for neural network layers such as the LSTM layer, there are multiple layers of parameters, but in order to enhance the generalizability of the model, we still use each layer of parameters as a single gene of the chromosome to participate in the crossover and mutation process; that is, some hidden layer may correspond to multiple chromosomal genes.

### 5.2. Performance Comparison among Three Algorithms

**Figure 4:**
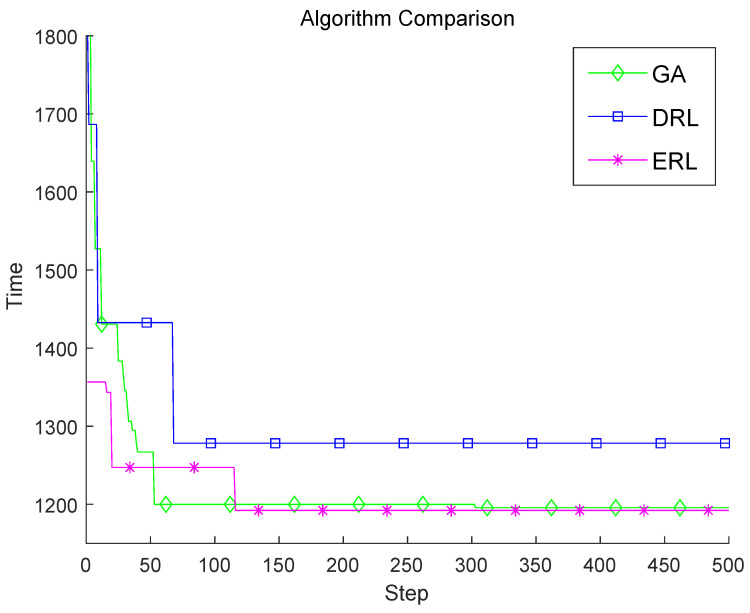
Comparison of the best results of different algorithms.

After 500 iterations, GA, DRL, and ERL obtain the optimal layout of sensors, the curves of the detection time of the layout of sensors by three algorithms is shown in Figure 4. As can be seen, the optimal layout of sensors by ERL has the shortest detection time compared to GA and DRL. From the perspective of the speed of the search process, GA has the highest rate of convergence, and DRL converges slowly.

Table 1 lists the average detection time by different layouts of sensors by three algorithms. As can be seen, the average detection time of deployed sensors by GA and ERL are 5975 (1195 × 5) min and 5960 (1192 × 5) min. Thus, it follows that GA and ERL outperform DRL.

### 5.3. Sensitivity Analysis

#### 5.3.1. Domain Knowledge with ERL

Experimental configurations in this and subsequent sections are shown in Table 2. In contrast, we extract multiple sets of solutions from our stochastic policy P(S|V) and choose the shortest one for the task without selecting nodes with probability. This method is called greedy selection.

**Figure 5:**
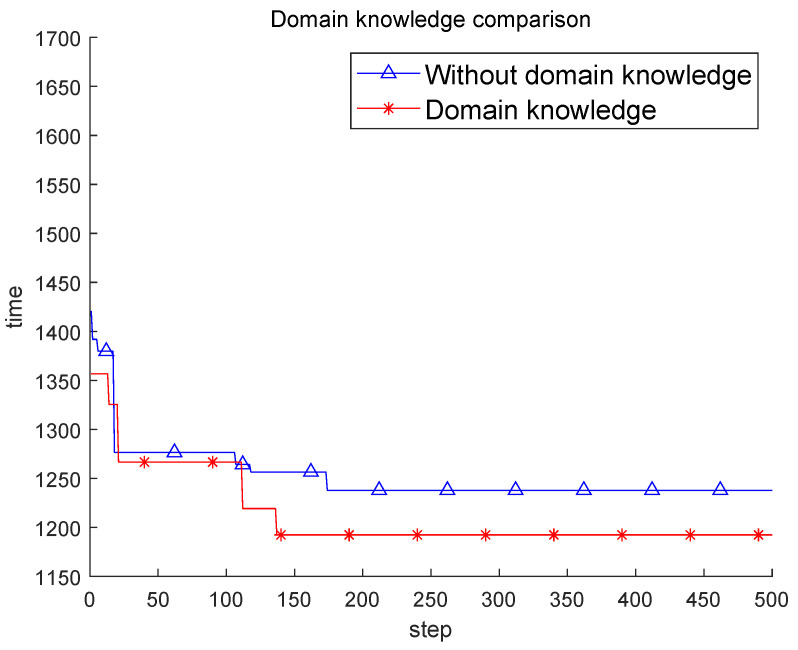
Domain knowledge comparison-based ERL.

In this part of the experimental comparison, we compare the ERL algorithm with and without domain knowledge, and the results are shown in Table 3. Except for whether the domain knowledge is added or not, two selection strategies and population evolution are included. The experimental results are shown in Figure 5. It can be seen that after adding domain knowledge, the search effect of the algorithm is obvious. The improvement not only greatly improves the results of the algorithm, but also requires fewer steps to reach the optimal results of the algorithm. It can be seen that after removing the domain knowledge, since only the index of the sensor is obtained, there is no understanding of the specific characteristics of each sensor. In this case, the search potential of the model at the later stage of the iteration becomes worse, and a good effect cannot be obtained.

#### 5.3.2. Nodes Selection Strategy with ERL

**Figure 6:**
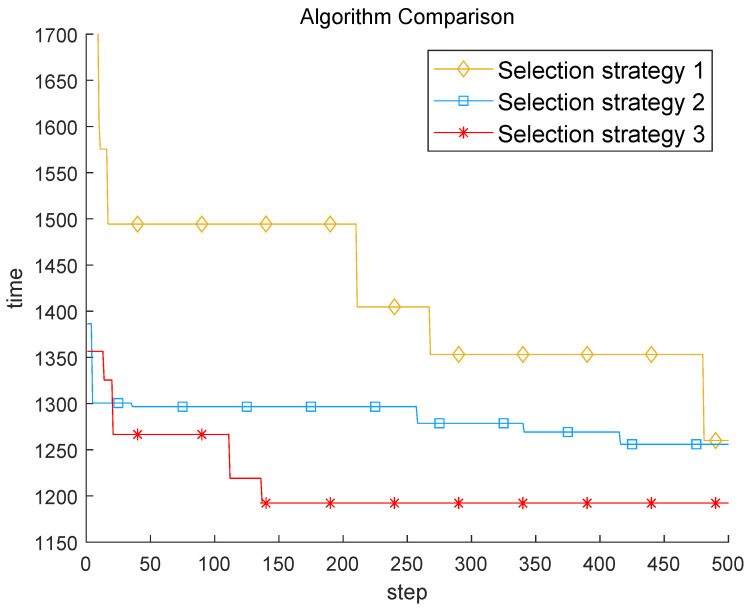
Selection strategy comparison based ERL.

The experimental results are shown in Figure 6. Selection strategy 1 uses multi-threaded search to explore multiple sets of feasible solutions to train the model at the same time, and uses greedy decoding for node selection, and only selects the node with the highest probability of being selected in each round of selection. Selection strategy 2 is the opposite of selection strategy 1. It uses probabilistic decoding, and randomly selects nodes according to the probability that the nodes selected in each round are selected, but only uses a single-threaded search. Strategy 3 is chosen to use both probabilistic decoding and multithreaded search. Greedy decoding is not a good choice for problems with high dimensional and nonlinear solution space. The first selection strategy has the worst effect, and the missing multi-threaded search in the second selection strategy is also critical. After adding the two selection strategies at the same time, the third selection strategy has a great improvement.

#### 5.3.3. Population Evolution with ERL

**Figure 7:**
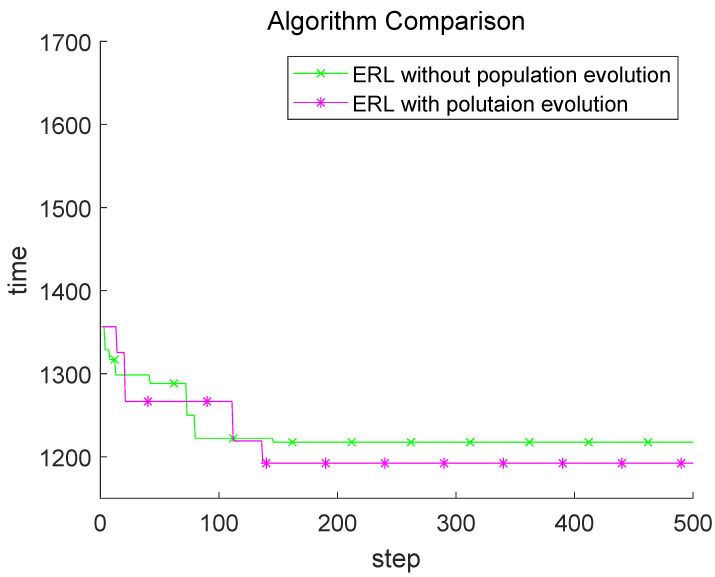
Population evolution comparison-based ERL.

The experimental results are shown in Figure 7. The cross-mutation operator will re-disrupt the model part in the convergence process. In this case, there may be no obvious difference in the initial stage, or even a slight decrease in efficiency. This can be seen from the comparison that the search ability of the later model will be greatly improved. It is precisely because of the addition of the cross mutation operator that the model has the ability to descend better [step100–step150].

## 6. Results and Discussion

To sum up, the ERL algorithm based on domain knowledge is feasible and efficient for SPP. Through the pointer network and attention mechanism, it is possible for the ERL algorithm to solve SPP. We conducted a comprehensive investigation of existing solutions to this problem and noticed that RL is a promising tool for solving this problem. However, none of the existing studies discusses this issue in WSN. Therefore, we are motivated to propose a customized ERL algorithm to solve this problem. We first use RL paradigm and pointer network individuals to build a framework for DRL to solve the problem of the optimal placement of sensors, and add evolutionary algorithms to the framework, and finally add domain knowledge to improve the search efficiency of the model. The improved algorithm surpasses the heuristic algorithm under the same conditions. As the first experiment, our work proved the feasibility and efficiency of applying ERL to sensor optimization problems.

## Figures and Tables

**Table 1:** Algorithm comparison.

Algorithm	GA	DRL	ERL
Average detection time/5 min	1195.606	1278.162	1192.254

**Table 2:** Different learning configurations.

Task Index	Domain Knowledge	Select with Probability	Multi-Threaded Search	EC Operator
*B*	No	Yes	Yes	Yes
$C_{1}$	Yes	No	Yes	Yes
$C_{2}$	Yes	Yes	No	Yes
*D*	Yes	Yes	Yes	No
For Comparison	Yes	Yes	Yes	Yes

**Table 3:** Algorithm comparison.

Task Index	Average Detection Time/5 min	Optimal Algebra
*B*	1237.8	174
$C_{1}$	1353.23	268
$C_{2}$	1255.95	416
*D*	1217.55	146
For Comparison	1192.25	137

## Data Availability

We use the open source software EPANET for simulating the effect of a contamination event distributed through WSN. The EPANET is a computer program performing an extended period simulation of hydraulic and water quality behavior. The download URL of EPANET is as follows: https://www.epa.gov/water-research/epanet (accessed on 8 March 2022). The network’s EPANET input files can be downloaded from the Exeter Centre for Water Systems(ECWS). The download URL of ECWS is as follows: http://emps.exeter.ac.uk/engineering/research/cws/ (accessed on 8 March 2022).

## References

[1] Grossman D.S., Slusky D.J. (2019). The impact of the Flint water crisis on fertility. Demography.

[2] Huang Z., Liu C., Zhao X., Dong J., Zheng B. (2020). Risk assessment of heavy metals in the surface sediment at the drinking water source of the Xiangjiang River in South China. Environ. Sci. Eur..

[3] Hu C., Dai L., Yan X., Gong W., Liu X., Wang L. (2020). Modified NSGA-III for sensor placement in water distribution system. Inf. Sci..

[4] Zhao Y., Schwartz R., Salomons E., Ostfeld A., Poor H.V. (2016). New formulation and optimization methods for water sensor placement. Environ. Model. Softw..

[5] Berry J., Hart W.E., Phillips C.A., Uber J.G., Watson J.P. (2006). Sensor placement in municipal water networks with temporal integer programming models. J. Water Resour. Plan. Manag..

[6] Propato M. (2006). Contamination warning in water networks: General mixed-integer linear models for sensor location design. J. Water Resour. Plan. Manag..

[7] Morais D.C., de Almeida A.T., Figueira J.R. (2014). A sorting model for group decision making: A case study of water losses in Brazil. Group Decis. Negot..

[8] Haghighi A., Asl A.Z. (2014). Uncertainty analysis of water supply networks using the fuzzy set theory and NSGA-II. Eng. Appl. Artif. Intell..

[9] Li M., Liu S., Zhang L., Wang H., Meng F., Bai L. (2012). Non-dominated sorting genetic algorithms-iibased on multi-objective optimization model in the water distribution system. Procedia Eng..

[10] Packer C., Gao K., Kos J., Krähenbühl P., Koltun V., Song D. (2018). Assessing generalization in deep reinforcement learning. arXiv.

[11] Lee K., Lee K., Shin J., Lee H. (2019). Network randomization: A simple technique for generalization in deep reinforcement learning. arXiv.

[12] Ouyang W., Wang Y., Han S., Jin Z., Weng P. Improving Generalization of Deep Reinforcement Learning-based TSP Solvers. Proceedings of the 2021 IEEE Symposium Series on Computational Intelligence (SSCI).

[13] Lu H., Zhang X., Yang S. A learning-based iterative method for solving vehicle routing problems. Proceedings of the International Conference on Learning Representations.

[14] Yolcu E., Póczos B. Learning local search heuristics for boolean satisfiability. Proceedings of the 33rd Conference on Neural Information Processing Systems (NeurIPS 2019).

[15] James J., Yu W., Gu J. (2019). Online vehicle routing with neural combinatorial optimization and deep reinforcement learning. IEEE Trans. Intell. Transp. Syst..

[16] Khadka S., Tumer K. (2018). Evolutionary reinforcement learning. arXiv.

[17] Drugan M.M. (2019). Reinforcement learning versus evolutionary computation: A survey on hybrid algorithms. Swarm Evol. Comput..

[18] Wang Z., Li H.X., Chen C. (2019). Reinforcement learning-based optimal sensor placement for spatiotemporal modeling. IEEE Trans. Cybernet..

[19] Maschler T., Savic D.A. (1999). Simplification of water supply network models through linearisation. Cent. Water Syst. Rep..

[20] Di Nardo A., Di Natale M., Giudicianni C., Greco R., Santonastaso G.F. (2018). Complex network and fractal theory for the assessment of water distribution network resilience to pipe failures. Water Sci. Technol. Water Supply.

[21] Wolsey L.A. (2020). Integer Programming.

[22] Brusco M.J., Stahl S. (2005). Branch-and-Bound Applications in Combinatorial Data Analysis.

[23] Hu C., Ren G., Liu C., Li M., Jie W. (2017). A Spark-based genetic algorithm for sensor placement in large scale drinking water distribution systems. Clust. Comput..

[24] Hu C., Li M., Zeng D., Guo S. (2018). A survey on sensor placement for contamination detection in water distribution systems. Wirel. Netw..

[25] Silver D., Schrittwieser J., Simonyan K., Antonoglou I., Huang A., Guez A., Hubert T., Baker L., Lai M., Bolton A. (2017). Mastering the game of go without human knowledge. Nature.

[26] Vinyals O., Fortunato M., Jaitly N. (2015). Pointer networks. arXiv.

[27] Bello I., Pham H., Le Q.V., Norouzi M., Bengio S. (2016). Neural combinatorial optimization with reinforcement learning. arXiv.

[28] Nazari M., Oroojlooy A., Snyder L.V., Takáč M. (2018). Reinforcement learning for solving the vehicle routing problem. arXiv.

[29] Chen X., Tian Y. Learning to perform local rewriting for combinatorial optimization. Proceedings of the 33rd Conference on Neural Information Processing Systems (NeurIPS 2019).

[30] Hajgató G., Paál G., Gyires-Tóth B. (2020). Deep Reinforcement Learning for Real-Time Optimization of Pumps in Water Distribution Systems. J. Water Resour. Plan. Manag..

[31] Mambretti S., Orsi E. (2016). Optimization of Pumping Stations in Complex Water Supply Networks through Evolutionary Computation Methods. J. Am. Water Works Assoc..

[32] Khadka S., Majumdar S., Nassar T., Dwiel Z., Tumer E., Miret S., Liu Y., Tumer K. Collaborative evolutionary reinforcement learning. Proceedings of the International Conference on Machine Learning (PMLR).

[33] Zhang K., Li Y., Wang J., Cambria E., Li X. (2021). Real-time video emotion recognition based on reinforcement learning and domain knowledge. IEEE Trans. Circuits Syst. Video Technol..

[34] Zheng Y., Chen H., Duan Q., Lin L., Shao Y., Wang W., Wang X., Xu Y. Leveraging Domain Knowledge for Robust Deep Reinforcement Learning in Networking. Proceedings of the IEEE INFOCOM 2021—IEEE Conference on Computer Communications.

